# Supplementary material for: Ethanolic Extract from Seed Residues of Sea Buckthorn (Hippophae rhamnoides L.) Ameliorates Oxidative Stress Damage and Prevents Apoptosis in Murine Cell and Aging Animal Models
Source: Foods. 2023 Sep 4;12(17):3322. doi: 10.3390/foods12173322 (PMC10487224; doi:10.3390/foods12173322)
Supplement: Supplementary file 1 [file foods-12-03322-s001.zip › foods-2572578-supplementary.pdf]

Supplemental Methods for proteomics

The whole tissue proteins of liver in group Control, Model, and HYD-SBSR-M were extracted using RIPA lysis buffer (RIPA; Millipore Sigma). The supernatant was collected for protein concentration detection using BCA kit (Thermo Scientific, USA) according to the manufacturer's instructions. The protein solutions were then reduced, alkylated, acetone precipitated, resuspended and digested with trypsin, and further desalted by Strata X C18 SPE column (Phenomenex) and vacuum-dried. Peptides were reconstituted and processed according to the manufacturer's protocol for TMT labeling using TMT kit (Thermo Scientific, USA). For HPLC fractionation, the tryptic peptides were fractionated into fractions by high pH reverse-phase HPLC using Thermo Betasil C18 column (5 μm particles, 10 mm ID, 250 mm length). Briefly, peptides were first separated with a gradient of 2% to 90% acetonitrile (pH 10.0) over 67 min into 50 fractions. Then, the peptides were combined into 20 fractions and dried by vacuum centrifuging. For LC-MS/MS analysis, the tryptic peptides were dissolved in 0.1% formic acid (solvent A), directly loaded onto a home-made reversed-phase analytical column. The gradient was comprised of an increase from 3% to 7% solvent B (99.9% acetonitrile and 0.1% formic acid) over 5 min, 7% to 16% in 31.5min, 16% to 25% in 8.5 min, 25% to 30% in 2min and climbing to 80% in 1 min then holding at 80% for 5 min, all at a constant flow rate of 300 nL/min. The electrospray voltage applied was 2.0 kV. The m/z scan range was 350 to 1600 for full scan, and intact peptides were detected in the Orbitrap at a resolution of 70000. The parent ions were fragmented by high-energy collision-induced dissociation (HCD) for peptide identification and relative quantitation of TMT reporter ions. The original mass spectrometry data were analyzed based on mouse (mmu) protein database of National Center for Biotechnology Information (NCBI, https://www.ncbi.nlm.nih.gov/protein). Proteome Discoverer software (PD) was used to search for database, calculate and analyze the generated original data of mass spectrometry detection in the above data using the Sequest algorithm. Pancreatin specific digestion was selected, and at most two missing sites were allowed. Cysiodoacetylation and TMT modification were set as fixed modification parameters, and Methionine oxidation and phosphorylation were set as variable modification parameters. The tolerance of parent ion mass was 15 ppm, and the tolerance of child ion mass was 0.02 Da. Select highly reliable peptides under the condition of less than 1% FDR as filtering parameters for protein qualitative identification, and select specific peptides for relative quantitative analysis of proteins between different samples.

Table S1. DEPs in the control group vs the model group.

Accession	Symbol	Gene_ID	Fold Change	p-value	Regulated type
NP_035174.1	Pck1	18534	1.196476906	0.044895219	up
XP_006505409.2	Cald1	109624	1.162208717	0.026263489	up
NP_598917.1	Actn1	109711	1.11087479	0.032342051	up
NP_062766.2	Cyp3a25	56388	1.329599939	0.039799223	up
NP_941055.1	Iars2	381314	1.112119326	0.007679524	up
NP_663544.1	Mat2a	232087	1.134124321	0.003772144	up
NP_775539.1	Eif5	217869	1.109341459	0.003506754	up
NP_001269991.1	Ewsr1	14030	1.107082486	0.010884367	up
NP_038506.2	Azgp1	12007	1.101673594	0.030858531	up
NP_035385.1	Rbp4	19662	1.14615057	0.027117863	up
NP_001292478.1	Apoa2	11807	1.205169363	0.014296952	up
NP_001034736.3	Gbp11	634650	2.262680229	0.029581723	up
NP_766548.2	Adck5	268822	1.10902612	0.032716781	up
NP_079656.1	Eef1e1	66143	1.163316017	0.03599657	up
XP_011240842.1	Ecsit	26940	1.12305538	0.010403512	up
NP_031849.1	Cyp4b1	13120	1.121462748	0.024020632	up
XP_006500001.1	Rapgef4	56508	1.19731233	0.048684413	up
NP_001019777.1	Pdp2	382051	1.247198101	0.038102003	up
NP_001292215.1	Mpdz	17475	1.113485121	0.017942267	up
NP_904335.1	ND3	17718	1.119346054	0.015278819	up
NP_001344215.1	Arih2	23807	1.10071892	0.005279585	up
XP_006518978.1	Farp1	223254	1.255319503	0.000803871	up
NP_001159842.1	Trmt112	67674	1.1282727	0.029472112	up
XP_006509312.2	Aga	11593	1.195095275	0.002335059	up
NP_598579.1	AI597479	98404	1.100362349	0.044736668	up
NP_938046.2	Nup188	227699	1.133084144	0.039905534	up
NP_001153723.1	Zfp281	226442	1.209486893	0.013891682	up
NP_077792.2	Cptp	79554	1.338578558	0.045988269	up
NP_001191841.1	Ptrhd1	69709	1.496887328	0.001554467	up
NP_032325.2	Dnajb3	15504	1.156464607	0.036652032	up
NP_031705.3	Cebpd	12609	1.111487013	0.024725712	up
XP_006501947.1	Rtca	66368	1.344942047	0.035087076	up
NP_031520.1	Ass1	11898	0.848903236	0.019529678	down
NP_620084.2	Mthfd1	108156	0.888727018	0.000769836	down
NP_663339.1	Akr1d1	208665	0.883269028	0.029646622	down
NP_058044.1	Prdx4	53381	0.851634272	0.002698275	down
NP_001177377.1	Ddc	13195	0.875717452	0.046432723	down
NP_080714.2	Ppa1	67895	0.886992915	0.009519333	down
NP_608219.1	Fdp	110196	0.854543004	0.040983967	down
NP_076012.3	Pecr	111175	0.886846262	0.012407512	down
NP_082129.2	Mettl7b	71664	0.851943969	0.006896277	down
NP_035212.2	Pigr	18703	0.868934766	0.020919521	down
NP_663540.1	Sd	231691	0.873081674	0.038754567	down
NP_001342297.1	Fubp1	51886	0.871088086	0.027701625	down
NP_036096.1	Psma4	26441	0.890432739	0.011514141	down
NP_080877.3	Hyi	68180	0.653576043	0.044009835	down

XP_017176596.1	Zbed5	71970	0.866735033	0.049608279	down
XP_006507601.1	Sult1a1	20887	0.797550451	0.043854619	down
XP_006524947.1	Cyp4f16	70101	0.002877691	0.001141909	down
NP_058578.3	Sart1	20227	0.866139541	0.025433312	down
NP_149026.1	Cdo1	12583	0.704729487	0.028029923	down
NP_001297558.1	Slc30a9	109108	0.868910998	0.045317215	down
XP_006527298.1	As3mt	57344	0.798286909	0.002592879	down
NP_778152.1	Coa6	67892	0.834243789	0.002686838	down
NP_081187.2	Spcs1	69019	0.002843197	0.000331937	down
NP_056569.2	Rbbp9	26450	0.023692525	0.005809394	down
NP_080512.1	Wdr48	67561	0.781278631	0.00044769	down
NP_653095.1	Slc6a13	14412	0.044924759	7.21067E-05	down
NP_666179.2	Cpped1	223978	0.875380914	0.03410881	down
NP_034918.1	Mecp2	17257	0.043030387	0.000139103	down
XP_006530945.1	Dync1li2	234663	0.047788496	3.53273E-06	down
NP_071711.2	Smoc1	64075	0.878008666	0.02166436	down
XP_017173320.1	Fgf1	14164	0.877138028	0.023323512	down
NP_035448.2	Apc	20219	0.722652915	0.018293645	down
XP_006519211.1	Cog3	338337	0.014060901	1.64726E-07	down
NP_001341907.1	Atp9a	11981	0.843727344	0.018510887	down
NP_001075444.1	Mfap1b	100034361	0.037688094	1.10484E-05	down
NP_035651.1	Sdc4	20971	0.03829752	7.07164E-05	down
NP_780659.2	Wdr18	216156	0.054916518	0.000125409	down
NP_001155203.1	Ang	11727	0.873653983	0.030085184	down
NP_081215.1	1810009N02Rik	69099	0.739017772	0.006780952	down
NP_082287.1	Tom1l1	71943	0.898718855	0.01163606	down
XP_006540702.1	Herc2	15204	0.005889454	0.000114486	down
NP_001239457.1	Pqbp1	54633	0.893920654	0.036970709	down
NP_001272911.1	Fam173a	214917	0.894509355	0.039310155	down
NP_001342620.1	Ttc17	74569	0.003238578	4.50144E-05	down
XP_006499057.1	Ptprj	19271	0.816980649	0.048732077	down
XP_011243917.1	Colec10	239447	0.856221315	0.017146441	down
NP_001163959.1	Tprkb	69786	0.87271046	0.042159892	down

Table S2. DEPs in the model group vs HYD-SBSR group

Accession	Symbol	Gene_ID	Fold Change	p-value	Regulated type
NP_032924.1	Por	18984	1.104265387	0.013078447	up
NP_075614.1	Kng1	16644	1.200405816	0.015781891	up
NP_001188399.1	Papss2	23972	1.117452956	0.007909189	up
NP_001239018.1	Rbmxl1	19656	1.173961458	0.013185876	up
NP_001160095.1	Rbmx	19655	1.59221364	0.008143902	up
NP_035212.2	Pigr	18703	1.198358739	0.0253557	up
NP_031434.3	Plin2	11520	1.953509627	0.019090408	up
NP_001289425.1	Gjb1	14618	1.385136084	0.045019565	up
NP_032943.2	Ppt1	19063	1.246775177	0.010597894	up
NP_035385.1	Rbp4	19662	1.12640839	0.033390243	up

NP_001153490.1	Tor1aip1	208263	1.177622828	0.002326725	up
NP_666326.1	Tat	234724	1.6964031	0.049359676	up
NP_598428.2	Pdk2	18604	1.142062672	0.013535784	up
NP_061289.1	Mpc1	55951	1.139598836	0.005620673	up
NP_032904.1	Serpinf2	18816	1.116111948	0.026717961	up
NP_035447.3	Khdrbs1	20218	1.104257877	0.00709463	up
NP_001269024.1	Tjp3	27375	1.117422285	0.025477532	up
NP_083296.2	Abcb8	74610	1.525065994	0.013692335	up
NP_666169.1	Dap	223453	1.151936363	0.029278439	up
NP_032707.2	Ndrg1	17988	1.330492804	0.007911365	up
XP_006522732.1	Snx29	74478	1.103800283	0.034484985	up
NP_001139276.1	Nucks1	98415	1.239670429	0.041192513	up
NP_001129558.1	Dpysl3	22240	1.142638003	0.009918168	up
NP_001277979.1	Gpcpd1	74182	1.144855437	0.04526522	up
NP_001184076.1	Slc4a4	54403	1.164202503	0.000320258	up
NP_001159882.1	Rbm3	19652	1.499248848	0.028628999	up
NP_080899.1	Nudt21	68219	1.10401125	0.007313954	up
NP_080242.1	2310039H08Rik	67101	1.125212477	0.03318895	up
XP_006533534.1	Pig	276846	1.407530482	0.012692448	up
XP_017177361.1	Ncstn	59287	1.134820729	0.008760607	up
NP_001298017.1	Pml	18854	1.198201236	0.022625405	up
NP_659165.1	Tmem150a	232086	1.130503048	0.00822564	up
NP_035523.1	Slc1a2	20511	1.285746512	0.038196307	up
NP_001075123.1	Tex264	21767	1.277014568	0.018567152	up
NP_001103618.1	Mgat1	17308	1.231706284	0.007763672	up
XP_006516163.1	Ylpm1	56531	1.103806613	0.01864596	up
NP_033907.1	C1qb	12260	1.218503996	0.040480112	up
XP_017172290.1	Dnajc21	78244	1.112557034	0.044059593	up
NP_082150.1	Csnk1d	104318	1.157362899	0.022872248	up
XP_017168046.1	Ctcf	13018	1.144241559	0.007509915	up
XP_017173103.1	Plin5	66968	1.14583904	0.02060669	up
NP_852076.1	Aftph	216549	1.177968526	0.014074481	up
NP_001073597.1	Rnps1	19826	1.147609648	0.015014516	up
NP_031825.2	Ctse	13034	1.358054447	0.038277319	up
NP_001161163.1	Sirt4	75387	1.171233982	0.018939048	up
NP_803178.2	Ofd1	237222	1.113923811	0.030181432	up
XP_006524868.1	Plin5	66968	1.19544951	0.000708142	up
NP_035551.1	Smpd1	20597	1.17426663	0.028275902	up
XP_006497658.1	Miga2	108958	1.100177543	0.013220406	up
XP_006501338.1	Arhgef11	213498	1.119897481	0.035790627	up
XP_006504481.1	Tpst1	22021	1.117836724	0.046572973	up
NP_001334585.1	Cdipt	52858	1.132562556	0.031166908	up
XP_011237506.1	Arl5a	75423	1.266558818	0.046032836	up
XP_017172882.1	Tsc2	22084	1.125052403	0.000122271	up
NP_733479.1	Nupl1	71844	1.102088193	0.014788653	up
NP_001030294.1	Golm1	105348	1.142384907	0.039364998	up
NP_666241.1	Pcif1	228866	1.237053981	0.031048683	up
NP_065606.2	Angptl4	57875	1.422254758	0.003570478	up

NP_032558.2	Epcam	17075	1.300372595	0.02054458	up
NP_031731.1	Cirbp	12696	1.133833036	0.012380989	up
XP_006503662.1	Gm17019	66773	1.357167878	0.045890888	up
NP_659043.1	Tmem63a	208795	1.107744851	0.035601215	up
NP_001010834.1	Slc10a5	241877	1.152991848	0.016029256	up
NP_033176.2	Selenbp1	20341	0.871230882	0.047421202	down
NP_034401.1	Gpd1	14555	0.865565417	0.038096529	down
NP_033399.1	Tgm2	21817	0.830232445	0.004987194	down
NP_080337.1	Ndufb8	67264	0.883792246	0.017273985	down
NP_663547.2	Gys2	232493	0.861297697	0.036629856	down
NP_001164471.2	Ahcyl2	74340	0.787519713	0.030894881	down
NP_036141.1	Eif2s3y	26908	0.860087984	0.003160655	down
XP_017176596.1	Zbed5	71970	0.76157783	0.00203081	down
NP_034490.1	Gstm5	14866	0.891853277	0.032309054	down
NP_033222.1	Slc16a1	20501	0.856058922	0.047848771	down
NP_001029101.1	Thnsl2	232078	0.814626275	0.049536756	down
NP_666262.2	Nrd1	230598	0.898610309	0.043321845	down
NP_079656.1	Eef1e1	66143	0.854203188	0.030938576	down
NP_076019.2	Pla2g12b	69836	0.21793034	0.033305208	down
NP_034317.1	Fcgr2b	14130	0.897953468	0.024864077	down
NP_001276406.1	Papss1	23971	0.823775587	0.024042376	down
NP_001344448.1	Xrcc5	22596	0.888310064	0.016099379	down
NP_001239582.1	Ecm1	13601	0.180531585	0.047787745	down
NP_598766.2	Enpp3	209558	0.780931595	0.002089257	down
NP_038941.1	Angptl3	30924	0.88777992	0.027427676	down
NP_001273097.1	Rbpm	19663	0.852915164	0.022661185	down
NP_758469.1	Eif2b5	224045	0.838992392	0.046525357	down
NP_001334347.1	Abcg8	67470	0.888829706	0.028252686	down
XP_006518978.1	Farp1	223254	0.834798262	0.019648087	down
NP_001036232.1	Proc	19123	0.83843475	0.035686021	down
NP_899098.2	Tefm	68550	0.835352608	0.022611185	down
XP_011236903.1	Osgepl1	72085	0.771133565	0.00221074	down
NP_073724.1	Scamp2	24044	0.874222189	0.015428382	down
NP_114090.1	Abcg5	27409	0.843376909	0.0191942	down
XP_006509312.2	Aga	11593	0.821681249	0.025899789	down
XP_006513123.1	Bcr	110279	0.71708395	0.013240822	down
XP_006538293.1	Dcaf12	68970	0.764632758	0.01650497	down
NP_073722.1	Polr1e	64424	0.877151646	0.013222445	down
NP_032417.3	Irf2	16363	0.548059401	0.009434334	down
NP_034886.1	Mafg	17134	0.875001078	0.019578963	down
NP_663512.2	Edem2	108687	0.872898218	0.035296972	down
NP_932140.1	Rcor1	217864	0.74155262	0.04329714	down
